# Preparation for mass gathering events from the perspective of a non-host country: the experience of Japan during the 2018 PyeongChang Olympics and Paralympic Winter Games

**DOI:** 10.5365/wpsar.2018.9.2.003

**Published:** 2019-02-08

**Authors:** Kazuaki Jindai, Takuya Yamagishi, Munehisa Fukusumi, Shingo Nishiki, Yusuke Kobayashi, Yusuke Matsui, Tamano Matsui, Kazunori Oishi

**Affiliations:** a Infectious Disease Surveillance Center, National Institute of Infectious Diseases, Tokyo, Japan.; b Field Epidemiology Training Program, National Institute of Infectious Diseases, Tokyo, Japan.

The World Health Organization recommends that countries or organizations that host mass gatherings plan ahead and prepare for possible public health events to ensure a safe environment for local residents, participants and travellers. (*1*) Public health events during mass gatherings can also affect non-host countries. There are numerous reports of the spread of infectious diseases by travellers returning from mass gatherings, (*2*) which can potentially pose the risk of an outbreak of new infectious diseases to travellers’ home countries. With more frequent travel across borders, it is prudent that non-host countries prepare for mass gathering events.

The 2018 PyeongChang Olympic Winter Games was held in the Republic of Korea between 9 February and 25 February 2018, followed by the Paralympic Games between 9 March and 18 March 2018. In both Games (hereinafter referred to as the Games), nearly 3000 athletes from 92 countries competed in 13 sports. Many travellers from Japan were expected to visit the Games. We conducted ad hoc event-based surveillance and risk assessments of Games-related public health events, especially infectious diseases outbreaks, which could affect Japanese athletes, travellers and residents in the Republic of Korea and which could have an influence on Japan. We described our methods and the lessons learnt through this project in this report.

**Fig. 1:**
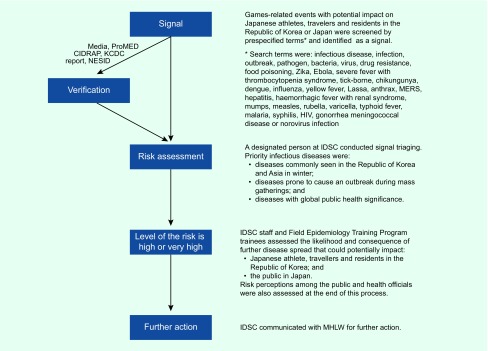
Algorithm of event-based surveillance and risk assessment of Games-related infectious disease events with potential impact on Japanese travellers and residents in the Republic of Korea during the 2018 PyeongChang Olympic and Paralympic Winter Games

CIDRAP: Center for Infectious Disease Research and Policy; IDSC: Infectious Disease Surveillance Center: National Institute of Infectious Diseases, Japan; KCDC: Korea Centers for Disease Control and Prevention; MHLW: Ministry of Health, Labour and Welfare, Japan; NESID: National Epidemiological Surveillance for Infectious Diseases, Japan.

One person was assigned to conduct event screening during weekdays using official and unofficial information sources (**Fig. 1**). During the Games, we identified priority infectious diseases to be monitored, such as diseases commonly seen in the Republic of Korea (e.g. mumps, hepatitis A and varicella); (*3*) diseases commonly seen in the winter in Asia (e.g. gastroenteritis and seasonal influenza); diseases that are prone to cause outbreaks during a mass gathering (e.g. meningococcal disease); and diseases with global public health significance (e.g. animal and human infection with avian influenza virus, measles, rubella and infection with multidrug-resistant bacteria). We screened reports from the media via Internet searches using the following pre-specified search terms:

- Infectious disease, food poisoning, infection, Zika, Ebola, severe fever with thrombocytopenia syndrome, chikungunya, dengue, pathogen, bacteria, virus, drug resistance, influenza, yellow fever, Lassa, anthrax, MERS, outbreak, hepatitis A, hepatitis, haemorrhagic fever with renal syndrome, mumps, measles, rubella, varicella, typhoid fever, malaria, syphilis, HIV, gonorrhea, meningococcal disease or norovirus infection.

We referred to the World Animal Health Information System, (*4*) ProMED, (*5*) the Center for Infectious Disease Research and Policy (*6*) and weekly reports from the Korea Centers for Disease Control and Prevention (KCDC) (*3*) to monitor events at the Games, using web-based automatic translation if written in the Korean language. We also screened weekly reports from the National Epidemiological Surveillance of Infectious Disease (NESID) in Japan (*7*) to search for indicators of potential disease importation from the Republic of Korea or disease spread in Japan. NESID includes more than 100 notifiable diseases and diseases on its sentinel surveillance system. (*8*) If we discovered pertinent signals, we contacted KCDC for signal verification. After verification, Infectious Disease Surveillance Center, Japan (IDSC) staff and Field Epidemiology Training Program (FETP) trainees assessed the likelihood and consequence of further spread of the event that could potentially impact on Japanese athletes, travellers, residents of the Republic of Korea and the public in Japan. The staff and trainees also assessed risk perception among Japanese nationals and among government officials of Japan to the event in the context of the Games. If the level of risk was high or very high, IDSC communicated with the Japanese Ministry of Health, Labour and Welfare (MHLW) for further action. The results were also shared with them at least weekly. We continued this activity for seven weeks: one week before, one week after and five weeks during the Games.

The mean number of signals identified per day was five. The average time required to triage all signals was 20 minutes per day. Five public health events underwent further risk assessments. One was a norovirus outbreak reported on 5 March 2018. (*9*) We determined that the risks of this event affecting Japanese travellers and residents in the Republic of Korea as well as spreading to Japan were low. There was a low probability that this event that would require an international response, given the low severity of the disease and quick response by the Korean Government. We also detected additional events of animal infection with avian influenza A(H5N6) virus; however, we considered the impact to be limited as transmission only occurred among poultry and wild birds. These events were shared with MHLW during the routine weekly meetings.

We conducted event-based surveillance (EBS) during a mass gathering event from the perspective of a non-host country. The focus was an event or signal that could pose any health risk to Japanese travellers and residents in the Republic of Korea during the Games. We also took into consideration the potential risk of such an event being imported to and spread in Japan.

A wide variety of infectious diseases have been associated with mass gatherings. (*2*) Diseases prone to spreading during a mass gathering should be prioritized for monitoring. The severity of diseases, the availability of treatments and public health control measures also need to be considered. The possibility of disease exportation from a non-host country to other countries is less likely in the early stage of a mass gathering event; therefore, we did not assess such risk. Instead, we highlighted attending athletes, travellers and residents in the Republic of Korea. This prioritization process required the knowledge of local context through consultation with KCDC through informal channels. Routine KCDC official reports based on existing indicator-based surveillance also provided relevant information and were helpful in understanding the situation. However, some pertinent information for events (e.g. detailed epidemiologic information of affected population) was not readily available from the reports. During the norovirus outbreak, additional detailed reports by KCDC of the outbreak played an important supplementary role in assessing the risk at the local level.

The primary concern when starting surveillance and risk assessment of the events associated with mass gathering was the burden on staff and other resources. Language barriers can lead to a greater burden on staff resources; however, we were able to reasonably understand information described in Korean through web-based automatic translation. One of our findings was that mass media in English language covered the events at the same time or quickly following reports published in the Korean media. Even if the lack of staff resources prevented close monitoring of Korean media, we believed that signals from English language media allowed the timely detection and response to events occurring at the Games. Another plausible concern for public health sectors in non-host countries is that additional time and resources are needed to implement an EBS system in another country. In this project, however, the ad hoc EBS system was an extension of an existing EBS system and did not result in additional financial and opportunity costs. (*10*) If the host country has an established, organized indicator-based surveillance system and is willing to share information in the system with non-host countries, the burden to the non-host country would be minimal.

If an event becomes an extended and serious threat to public health, prompt upscaling of the response is warranted. (*11*) As emphasized in the Asia Pacific Strategy for Emerging Disease and Public Health Emergencies, (*12*) having surge capacity to expand routine EBS and risk assessment is imperative to respond to an outbreak during large mass gatherings.

It seems feasible to apply similar methods during other mass gatherings, although limitations should be considered before planning a similar EBS and risk assessment system. First, successful EBS and risk assessment depend on the infrastructure of the host country and its information-sharing system. Second, disease prioritization for EBS and risk assessment during mass gatherings needs the understanding of local disease epidemiology and the context of the host country. Third, translations by web-based automatic translation systems can be inaccurate; (*13*) although the information in the media reports in English aligned with the original Republic of Korea reports.

We described our experience in EBS and risk assessment during a mass gathering from the perspective of a non-host country. Building in-country EBS, a risk assessment system and establishing lines of communication with host countries before the event are of critical importance for successful preparation. Regional networks can help to establish and maintain communication with host countries. Our experience could be a meaningful model for non-host countries to prepare and enhance EBS for mass gatherings held in other countries.
